# Global research trends in metabolism-related intraocular malignancies: a multi-database bibliometric analysis and cross-validation study

**DOI:** 10.3389/fmolb.2025.1683864

**Published:** 2025-09-09

**Authors:** Jianhao Bai, Zhongqi Wan, Zhiyong Wu, Qing Peng

**Affiliations:** ^1^ Department of Ophthalmology, Shanghai East Hospital, Tongji University School of Medicine, Shanghai, China; ^2^ Department of Ophthalmology, Shanghai Tenth People’s Hospital Affiliated to Tongji University, Tongji University School of Medicine, Shanghai, China; ^3^ Department of Ophthalmology, Shanghai Heping Eye Hospital, Shanghai, China

**Keywords:** intraocular malignancies, metabolism, bibliometric analysis, uveal melanoma, precision oncology

## Abstract

**Objective:**

To systematically characterize the global research landscape of metabolism-related intraocular malignancies and to validate the robustness of findings through a multi-database comparative approach.

**Methods:**

Publications from January 1, 1990, to July 31, 2025, were retrieved from the Web of Science Core Collection (WoSCC). To ensure the stability and generalizability of results, equivalent searches were performed in Scopus and PubMed, applying the same keyword set, time frame, and eligibility criteria. Bibliometric analyses were conducted using VOSviewer, CiteSpace, and GraphPad Prism to evaluate publication trends, geographic and institutional contributions, journal and author influence, keyword co-occurrence, co-citation patterns, and emerging research fronts. Cross-database validation assessed concordance in temporal trends, thematic focuses, and country rankings.

**Results:**

A total of 1,745 WoSCC publications were included, authored by researchers from 69 countries. Global output has increased markedly since 2010, peaking in 2021. Uveal melanoma consistently emerged as the dominant intraocular tumor type in metabolic research. Major thematic clusters encompassed oxidative stress, apoptosis, hypoxia, lipid metabolism, and metabolic reprogramming, with recent shifts toward long noncoding RNA, immune infiltration, and metabolomics, signaling a transition to precision oncology. Importantly, multi-database validation demonstrated high concordance in annual publication trends, as well as strong overlap in top keywords and stability in geographical and disease foci.

**Conclusion:**

This study provides a multi-database bibliometric assessment of metabolism-related intraocular malignancy research, with offering a reliable foundation for guiding future basic and translational research in ocular oncology.

## Introduction

Intraocular malignancies constitute a heterogeneous group of primary or secondary neoplasms originating within the eye, encompassing retinoblastoma, uveal melanoma (UM), intraocular lymphoma, and choroidal metastases (Trinh et al., 2004; Goldberg et al., 2007; Benavente and Dyer, 2015; Mathis et al., 2019; Nature Reviews Disease Primers, 2020). Although these tumors are relatively uncommon compared to other systemic malignancies, they hold substantial clinical significance due to their potential to induce irreversible vision loss, ocular destruction, and even life-threatening metastasis (Dennis et al., 2025). Retinoblastoma represents the most prevalent pediatric intraocular malignancy (Yanagihara et al., 2025), whereas UM is the most common intraocular cancer in adults (Carvajal et al., 2023). Despite advancements in diagnostic imaging and localized treatment modalities such as enucleation, brachytherapy, and laser therapy, the prognosis for metastatic UM remains dismal, with a 5-year survival rate of less than 15% (Nathan et al., 2021; Hassel et al., 2023). Similarly, recurrent or refractory retinoblastoma and aggressive ocular lymphomas continue to present formidable clinical challenges (Fabian et al., 2020). These limitations highlight the critical need for novel diagnostic markers and therapeutic strategies, informed by a more profound understanding of ocular tumor biology.

In recent years, metabolic reprogramming has been recognized as a hallmark of cancer and a central feature of tumor pathogenesis (Yang et al., 2020; Jin et al., 2023; Lin et al., 2024). Malignant cells undergo significant metabolic alterations to satisfy the demands of rapid proliferation, invasion, immune evasion, and therapy resistance. These changes encompass not only glycolytic activation, known as the Warburg effect (Fendt, 2024), but also mitochondrial dysfunction, lipid biosynthesis, glutamine dependence, and redox imbalance (Faubert et al., 2020; Xia et al., 2021). Within the context of intraocular malignancies, accumulating evidence indicates that metabolic pathways are crucial in tumor initiation, progression, and therapeutic response. For example, altered oxidative phosphorylation and fatty acid metabolism have been implicated in the aggressiveness of UM and the modulation of its immune microenvironment (Song et al., 2024; Xu et al., 2024; Sun et al., 2025). Additionally, retinal tumors such as retinoblastoma may exploit glycolytic and hypoxic pathways to sustain survival within the unique metabolic niche of the eye (Babu et al., 2022).

Despite the increasing acknowledgment of metabolic dysregulation within ocular oncology, current research remains disjointed. The majority of existing studies concentrate on specific tumor types or isolated metabolic pathways, lacking a comprehensive synthesis of the global research landscape (de Bruyn et al., 2023). The field is further limited by insufficient cross-institutional collaboration and the underexploration of translational applications. Additionally, the incorporation of emerging methodologies such as metabolomics, metabolic imaging, and multi-omics platforms into ocular tumor research is still nascent.

In light of these challenges and opportunities, a systematic bibliometric analysis is necessary to chart the evolution, trends, and frontiers of metabolism-related research in intraocular malignancies. By quantitatively assessing global publication output, citation impact, authorship networks, and thematic developments over the past three decades, this study seeks to offer a comprehensive overview of this interdisciplinary field. Such insights may not only elucidate existing research gaps but also inform future endeavors in the development of metabolism-targeted diagnostics and therapies for intraocular tumors.

## Materials and methods

### Data source and search strategy

This bibliometric study was conducted based on publications retrieved from the Web of Science Core Collection (WoSCC) database. On July 31, 2025, we systematically searched for all publications related to metabolism and intraocular malignancies published between January 1, 1990, and July 31, 2025. The search strategy combined disease-specific terms with metabolism-related terms using Boolean operators. The complete search formula is provided in Supplementary Table S1.

### Inclusion and exclusion criteria

**FIGURE 1:**
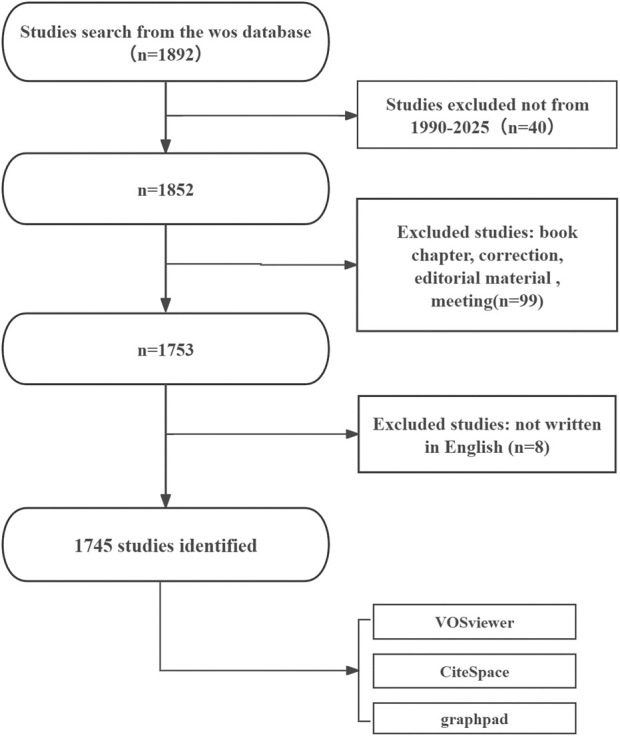
Flowchart of the literature screening and selection process.

Studies were considered eligible if they met all of the following criteria: (1) full-text publications directly addressing metabolism in intraocular malignancies; (2) original research articles or review papers written in English; and (3) published within the defined time frame of January 1, 1990, to July 31, 2025. Exclusion criteria were as follows: (1) studies deemed unrelated to the topic after title and abstract screening; and (2) document types such as meeting abstracts, editorials, letters, news items, and brief communications. Duplicate records were removed prior to analysis to ensure data integrity and consistency. Two independent reviewers screened the titles and abstracts of all retrieved records to determine relevance. Studies were excluded if they did not address both intraocular malignancies and metabolic processes. Disagreements were resolved through discussion, and a third reviewer was consulted if necessary. The literature screening process is illustrated in Figure 1. An initial search of the WoSCC database yielded 1,892 records. After removing 40 records outside the time window, 1,852 remained. A further 99 records were excluded due to their document type (e.g., book chapters, corrections, editorial materials, or meeting abstracts), and 8 non-English publications were removed. The final dataset comprised 1,745 eligible publications, which were subsequently subjected to bibliometric and visualization analyses using VOSviewer, CiteSpace, and GraphPad Prism. As the data collection date was July 31, 2025, we acknowledge that publications from the latter half of 2025 may not have been fully indexed. As such, 2025 data may be subject to underestimation.

### Indicators and visualizations

A comprehensive set of bibliometric indicators was assessed, including annual publication counts, total citations, average citations per publication, and h-index. The h-index was used to evaluate author impact, defined as the number of publications (h) that have received at least h citations. Country- and institution-level performance was evaluated by publication volume, total citations, average citation impact, and betweenness centrality within collaboration networks. Betweenness centrality reflects how often a node acts as a bridge between other nodes in the collaboration network. Journal-level influence was determined by publication count, impact factor, quartile ranking, and co-citation frequency. Author productivity and the underlying intellectual structure of the field were explored via author collaboration networks, co-citation mapping, and clustering of core contributors. Keyword co-occurrence networks and cluster analyses were employed to identify research hotspots and track thematic evolution, while burst detection analysis was used to identify emerging research fronts.

### Data analysis and visualization

Quantitative, network-based, and structural analyses were performed using established bibliometric tools. VOSviewer (version 1.6.18) was applied to construct and visualize author collaboration networks, institutional co-authorship networks, journal distribution patterns, and keyword co-occurrence maps. CiteSpace (version 6.2.4R) was employed for co-citation reference analysis, keyword clustering, timeline mapping, and burst detection of both keywords and references. The main parameters for CiteSpace were: time slicing from 1990 to 2025 with 1-year intervals; term sources including titles, abstracts, author keywords, and cited references; Top N = 50 items per slice; and clustering based on the log-likelihood ratio (LLR) algorithm. GraphPad Prism (version 8.0.2) was used to generate statistical plots illustrating annual publication trends, national outputs, and proportional contributions by country.

### Multi-database validation

To verify the robustness and comprehensiveness of the results obtained from the WoSCC dataset, an additional search was conducted in Scopus and PubMed on the same retrieval date (July 31, 2025). Equivalent search strategies, including the same set of keywords, Boolean operators, and time frame (January 1, 1990–July 31, 2025), were adapted to the syntax requirements of each database. The inclusion and exclusion criteria applied to WoSCC were consistently implemented for the Scopus and PubMed searches to ensure comparability.

For each supplementary database, the total number of retrieved records, annual publication trends, top contributing countries, and high-frequency author keywords were extracted and compared with the primary WoSCC dataset. The degree of consistency in temporal trends was assessed using Pearson correlation analysis of annual publication counts across databases. Overlapping and unique records were identified through bibliographic matching based on title, authors, and publication year. This cross-database comparison was used to confirm whether the thematic focuses, geographical distribution, and temporal patterns observed in the WoSCC-based analysis were reproducible in other major bibliographic sources.

### Ethical considerations

This study did not involve human participants, animals, or clinical interventions. All data used were obtained from publicly available bibliographic databases; therefore, no ethical approval was required.

## Results

### Publication trends

**FIGURE 2:**
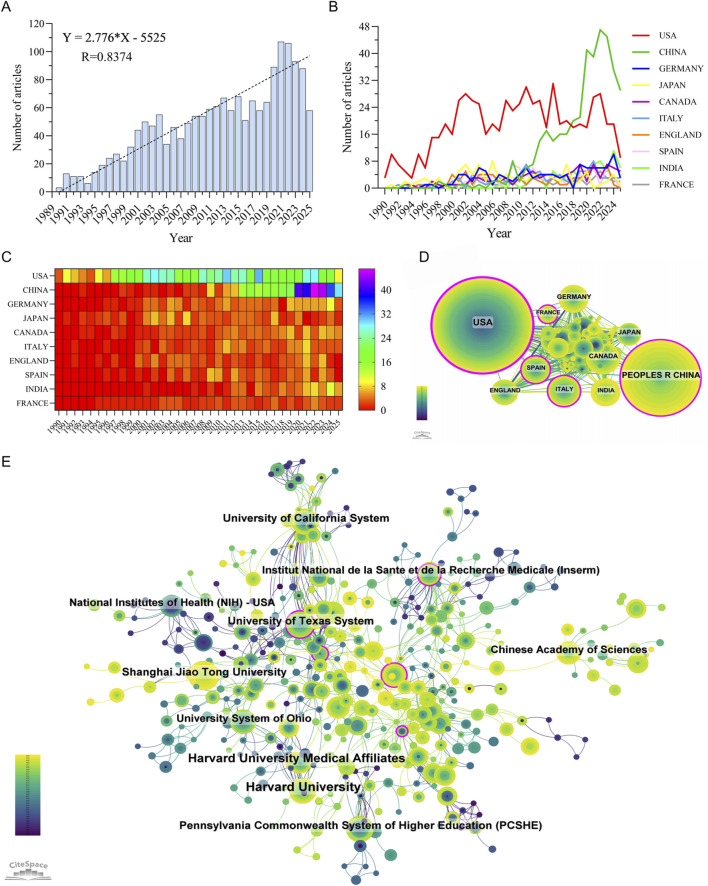
Publication trends and global contributions in the field of metabolism-related intraocular tumor research. **(A)** Annual number of publications from 1990 to 2025. **(B,C)** Line chart **(B)** and heatmap **(C)** illustrating the annual publication volume of the top 10 countries. **(D)** International collaboration network among countries, with node size representing publication volume and line thickness indicating collaboration strength. **(E)** Top contributing institutions and their collaboration network, highlighting institutional clusters and cross-national partnerships.

Between January 1, 1990, and July 31, 2025, a total of 1,745 publications related to metabolism and intraocular tumors were retrieved from the WoSCC database, including 1,483 original articles and 262 reviews, contributed by 9,350 authors from 2,052 institutions across 69 countries and regions. As shown in Figure 2A, the annual number of publications exhibited a steady upward trend over the past 35 years (R = 0.8374), which can be divided into three distinct phases. During 1990-1996, the field remained in its infancy with slow growth. From 1997 to 2019, the number of publications increased rapidly, reflecting growing interest in tumor metabolism. Since 2019, research activity has further intensified, reaching a peak in 2021. It should be noted that the apparent decline in publication output in 2025 may be attributable to ongoing indexing delays, as the data were retrieved in late July 2025. Therefore, the 2025 data likely do not represent the full year and should not be interpreted as a definitive downward trend.

### Global and Institutional contributions to the field

A total of 69 countries and regions have contributed to research on metabolism in intraocular malignancies, forming a globally distributed but uneven academic landscape. As illustrated in Figures 2B,C, the United States has maintained a leading role throughout the entire study period, contributing 666 publications (38.17%), with a citation count of 48,850 and the highest citation-per-publication ratio of 73.35 (Supplementary Table S2). This indicates not only the quantity but also the sustained quality and influence of U.S.-based research.

In recent years, China has emerged as a major contributor, ranking second in both publication volume (389 articles, 22.29%) and total citations (8,457). However, its citation-per-publication ratio (21.74) remains significantly lower than those of most high-income countries, reflecting a gap in research influence and visibility despite rapid growth. The United Kingdom, Canada, Germany, and Japan, while contributing fewer articles, demonstrated outstanding average citation rates (e.g., United Kingdom: 80.96, Canada: 59.96), suggesting a focus on high-impact, targeted studies.

As shown in Figure 2D, international collaboration networks are dominated by a few central nodes, with the United States displaying the highest betweenness centrality (0.47), underscoring its pivotal role as a global connector. The U.S. maintains strong collaborative links with European countries such as Germany, France, and England, while China tends to collaborate more with regional partners including Japan, India, and Canada. Despite increasing cross-national engagement, most countries still exhibit a tendency toward intra-national institutional collaboration, highlighting a need to further internationalize research efforts.

Institutional-level analysis (Figure 2E; Supplementary Table S3) further supports the dominance of U.S. academic centers. Among the top 10 most productive institutions, 7 are based in the United States, including Harvard University (57 publications, 5,208 citations, 91.37 citations/article) and the University of California System (33 publications, 3,380 citations, 102.42 citations/article), both reflecting exceptional research productivity and impact. The Chinese Academy of Sciences and Shanghai Jiao Tong University represent China’s leading institutions, but their average citation rates (38.04 and 36.58, respectively) lag behind their U.S. counterparts. Collaboration networks at the institutional level reveal tight-knit clusters primarily centered within national boundaries, with limited cross-national institutional partnerships. This pattern suggests that while global participation in this field is expanding, true international research integration remains insufficient. Bridging this divide—especially between high-output but less-central institutions and core global hubs—could accelerate innovation and knowledge transfer across borders.

### Journal analysis

**FIGURE 3:**
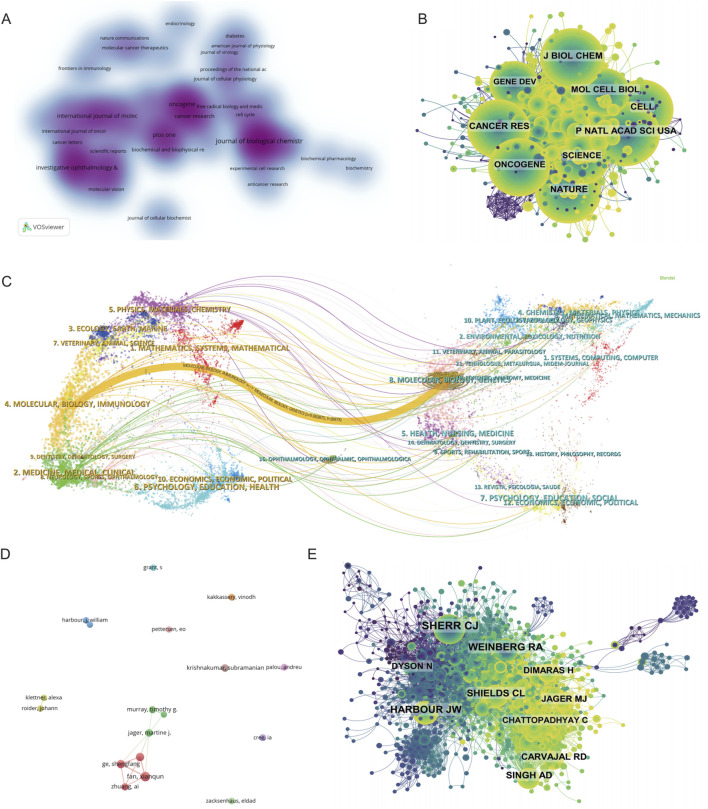
Mapping the Research Landscape of Metabolism-Related Intraocular Tumors: Publication Density, Journal Networks, and Author Collaborations. **(A)** Density map of journal publications in the field of metabolism related to intraocular tumors. **(B)** Co-citation network of the top co-cited journals. **(C)** Dual-map overlay of journals related to metabolism in intraocular tumors, where colored tracks represent citation pathways, with citing journals on the left and cited journals on the right. **(D)** Author collaboration network chart. **(E)** Author co-citation network map illustrating the most frequently co-cited authors.

A comprehensive analysis of journal distribution revealed both the productivity and influence of scientific publications within the field of metabolism and intraocular malignancies. As shown in Supplementary Table S4, the top 10 most productive journals collectively accounted for 19.88% of the total 1,745 articles. The Journal of Biological Chemistry ranked first with 66 publications (3.78%), followed by Oncogene (38, 2.18%), International Journal of Molecular Sciences (33, 1.89%), and PLOS ONE (33, 1.89%). Among them, Cancer Research had the highest IF (16.6) and was classified as a Q1 journal, highlighting its relevance in high-impact translational oncology research. Notably, 80% of the top 10 journals were classified in Q1 or Q2 categories, reflecting the overall quality and recognition of this research domain. The journal density visualization generated by VOSviewer (Figure 3A) identified several high-density clusters, indicating concentrated publication activity. These included journals focusing on molecular biology and metabolism (J Biol Chem, Cancer Research, Oncogene), ophthalmology (Investigative Ophthalmology & Visual Science, Molecular Vision), and interdisciplinary platforms (PLOS ONE, IJMS, Scientific Reports). This pattern reflects the cross-disciplinary nature of the field, integrating metabolic mechanisms, tumor biology, and ophthalmic pathology.

In terms of academic influence, Supplementary Table S5 lists the top 10 most co-cited journals, led by Proceedings of the National Academy of Sciences of the United States (PNAS) with 1,076 citations, followed closely by Journal of Biological Chemistry (1,045) and Nature (1,031). Highly influential general science journals such as Nature, Science, and Cell were frequently co-cited despite not being field-specific, indicating their critical role in supporting foundational theories and methodologies in this field. 90% of the most co-cited journals belonged to Q1/Q2, further underscoring the dependence on high-quality literature. The journal co-citation network shown in Figure 3B further supports the central influence of high-impact, widely referenced journals such as J Biol Chem, PNAS, Nature, Cancer Research, and Oncogene. These journals formed the structural core of the knowledge network, indicating their foundational status within the field’s theoretical framework. Dense citation linkages among these journals suggest a coherent and mature citation ecosystem that supports both experimental and translational studies.

Lastly, the dual-map overlay of journals (Figure 3C) provided a macro-level visualization of knowledge flow between citing and cited disciplines. The dominant citation path extended from publications in “Molecular/Biology/Immunology” and “Medicine/Medical/Clinical” journals on the left to “Molecular Biology/Genetics” and “Health/Nursing/Medicine” journals on the right. This indicates that authors in this field mainly publish in biomedical and clinical journals while drawing heavily from foundational research in molecular biology, genetics, and oncology. Secondary knowledge flows were observed from physics and chemistry journals, suggesting additional input from fields such as biomaterials and nanotechnology. Collectively, these findings demonstrate that the field of metabolism-related intraocular tumor research is rooted in high-quality, interdisciplinary literature. It draws extensively from both fundamental biological sciences and clinical medicine, while increasingly incorporating tools and knowledge from translational research and bioengineering domains.

### Author and Co-Cited author analysis

A total of 102 articles were published by the top 10 most productive authors, accounting for 5.85% of all publications in this field. Among them, Fan Xianqun ranked first with 16 papers, followed by Ge Shengfang and Jia Renbing with 12 publications each, and Jager Martine J. with 10 publications (Supplementary Table S6). As illustrated in the author collaboration network (Figure 3D), a strong collaboration cluster centered on Chinese scholars, including Fan, Ge, and Zhuang Ai, was observed. This cluster exhibits high internal connectivity, suggesting the presence of a cohesive research team that contributes significantly to the field. In contrast, other active authors such as Krishnakumar Subramanian and Kakkassery Vinodh appeared relatively isolated, indicating more independent research trajectories.

Co-citation analysis further elucidates the intellectual base of the field. A total of 44 authors were cited over 100 times, highlighting their substantial influence (Figure 3E). Sherr CJ was the most frequently co-cited author (189 citations), followed by Harbour JW (126) and Weinberg RA (124). These scholars are known for their foundational work in molecular oncology and tumor biology, and their prominence in the network reflects the translational nature of metabolic research in ocular malignancies. The co-citation network (Figure 3E), constructed using CiteSpace, reveals a dense and interconnected structure, with several large nodes representing key contributors such as Shields CL, Singh AD, Jager MJ, and Carvajal RD, all of whom are closely linked with clinical and translational studies in UM and retinoblastoma.

### Co-cited references

**FIGURE 4:**
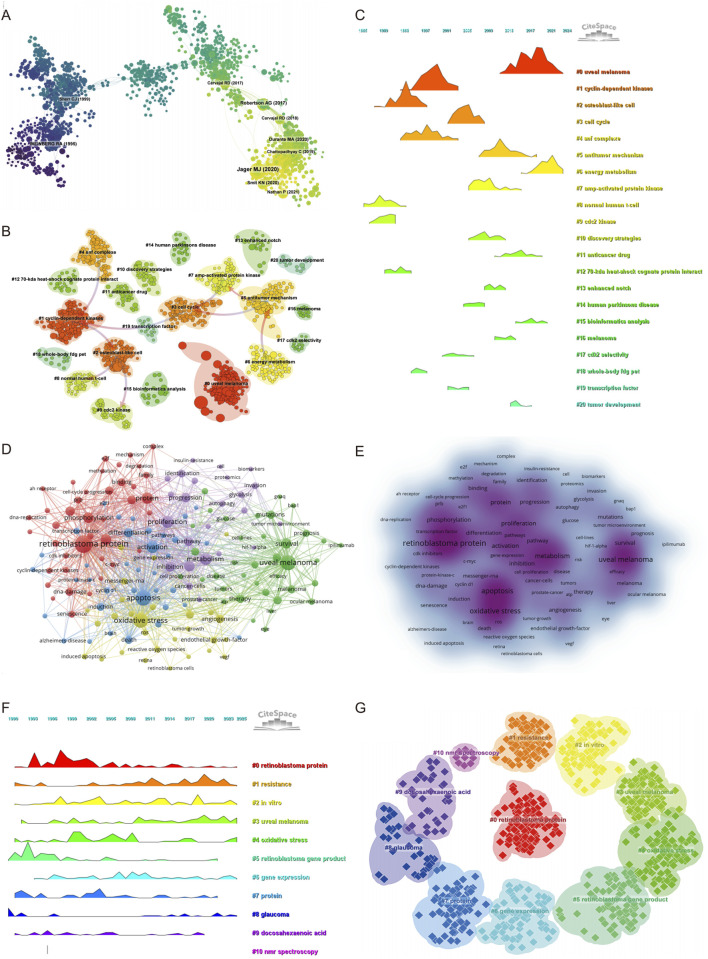
Keyword co-occurrence, clustering, and thematic evolution. **(A)** Co-cited literature network map showing foundational studies in metabolism and intraocular tumors. **(B)** Cluster analysis of co-cited references. **(C)** Temporal distribution of co-cited references illustrates the evolution of research topics over time. **(D)** Network diagram highlighting high-frequency keywords. **(E)** Keyword density map illustrating the concentration of key topics. **(F)** The temporal heatmap displays the progression of key research areas from 1990 to 2025. Each color-coded cluster represents a distinct research theme. **(G)** Clustering analysis of research hotspots in metabolism related to intraocular tumors.

Co-citation analysis provides insight into the foundational knowledge structure and emerging intellectual bases of a research field (Supplementary Table S7). As shown in the co-citation network (Figure 4A), a total of 1,547 references formed a network with 5,346 links, indicating a well-developed body of interlinked literature. The clustering analysis (Figure 4B) further identified distinct thematic clusters, with Cluster #0 labeled “uveal melanoma” being the largest and most recent, highlighting that current research on intraocular tumors and metabolism is predominantly centered on UM, particularly in relation to its molecular classification and metabolic reprogramming. Temporal visualization via the reference timeline (Figure 4C) reveals the evolution of research foci. Early clusters such as “cyclin dependent kinases” (Cluster #1), “osteoblast like cells” (Cluster #2), and “cdc2 kinase” (Cluster #9) indicate that foundational work initially emphasized classical cell cycle regulation and stress response mechanisms. In the intermediate phase, topics like “cell cycle” (Cluster #3), “AMP-activated protein kinase” (Cluster #7), and “discovery strategies” (Cluster #10) gained prominence, suggesting a transition toward metabolic pathway regulation and therapeutic exploration. The most recent clusters—such as “uveal melanoma” (Cluster #0), “antitumor mechanism” (Cluster #5), “energy metabolism” (Cluster #6), and “bioinformatics analysis” (Cluster #15)—reflect a growing emphasis on the metabolic and molecular basis of ocular tumors and precision-targeted therapy.

The most frequently co-cited reference was a landmark study published in Cancer Cell, titled “Integrative Analysis Identifies Four Molecular and Clinical Subsets in Uveal Melanoma.” This study performed a comprehensive multi-omics analysis of 80 UM samples and identified four distinct subtypes characterized by unique chromosomal alterations, immune microenvironment profiles, and metabolic signatures. Notably, alterations in chromosome 3 and BAP1 expression status, along with changes in oxidative phosphorylation and glycolysis-related pathways, were found to be strongly associated with prognosis. This work has profoundly influenced current understanding of the molecular heterogeneity of UM and underlines the importance of metabolism-related classification strategies. Its prominence in the co-citation network validates our bibliometric findings, emphasizing that the intersection of metabolism and intraocular malignancy, particularly in UM, represents a central and evolving theme in the field.

The co-citation landscape underscores a clear intellectual trajectory: from foundational studies on cell cycle and kinase regulation to recent integrative approaches focusing on molecular subtyping, immune modulation, and metabolic reprogramming in UM. These findings support the conclusion that metabolic and molecular investigations of intraocular tumors, especially UM, are at the forefront of contemporary research and likely to shape future therapeutic strategies.

### Keyword Co-occurrence and clustering analysis

A total of 165 keywords with a minimum occurrence of 14 times were included to construct the co-occurrence network. The top 20 high-frequency keywords are listed in Supplementary Table S8, with “retinoblastoma protein” (n = 320), “apoptosis” (n = 236), “uveal melanoma” (n = 226), and “oxidative stress” (n = 211) ranking highest. Notably, metabolism-related terms such as “metabolism” (n = 139), “proliferation” (n = 132), “activation” (n = 130), and “hypoxia” (n = 102) also appeared with high frequency, suggesting their relevance in ocular tumor research.

The keyword co-occurrence network was grouped into five clusters (Figure 4D), each representing a major thematic focus. Cluster 1 (red) centers on the molecular biology of retinoblastoma protein, including terms like “pRb (retinoblastoma tumor suppressor protein),” “CDK inhibitor,” “DNA damage,” and “E2F,” reflecting interest in tumor suppressor mechanisms and transcriptional regulation. Cluster 2 (green) is dominated by terms related to UM and clinical therapeutics, such as “mutation,” “BAP1,” “GNAQ,” “prognosis,” and “immunotherapy,” highlighting growing attention on molecular markers and treatment resistance in intraocular malignancies. Cluster 3 (blue) focuses on apoptosis and inflammatory signaling, with keywords like “cell proliferation,” “cyclin D1,” “TGF-beta,” and “tumorigenesis,” indicating research interest in cell death pathways. Cluster 4 (yellow) emphasizes oxidative stress and angiogenesis, including “NF-kappa B,” “VEGF,” “hydrogen peroxide,” and “photodynamic therapy,” revealing exploration of tumor microenvironment and redox biology. Cluster 5 (purple) centers on metabolic adaptation and tumor progression, including “autophagy,” “glycolysis,” “hypoxia,” “migration,” “proteomic,” and “tumor microenvironment,” underscoring the increasing integration of metabolomics into ocular tumor research. Additionally, the keyword density distribution (Figure 4E) further highlights the intensity of research focus across clusters.

The timeline visualization (Figure 4F) and cluster view (Figure 4G) reveal the temporal evolution and structural relationships of these keyword themes. Cluster #0″retinoblastoma protein” emerged early and remained a sustained research hotspot, while clusters #3″uveal melanoma” and #4″oxidative stress” showed continuous growth in recent years. Emerging topics such as cluster #5″retinoblastoma gene product,” #8″glaucoma,” and #9″docosahexaenoic acid (omega-3 fatty acid involved in lipid metabolism)” have gained traction after 2015, indicating a shift toward metabolite-specific investigations. Furthermore, cluster #10″NMR spectroscopy,” though with limited volume, represents the technical advancement in metabolic profiling methods.

### Emerging trends and new developments

**FIGURE 5:**
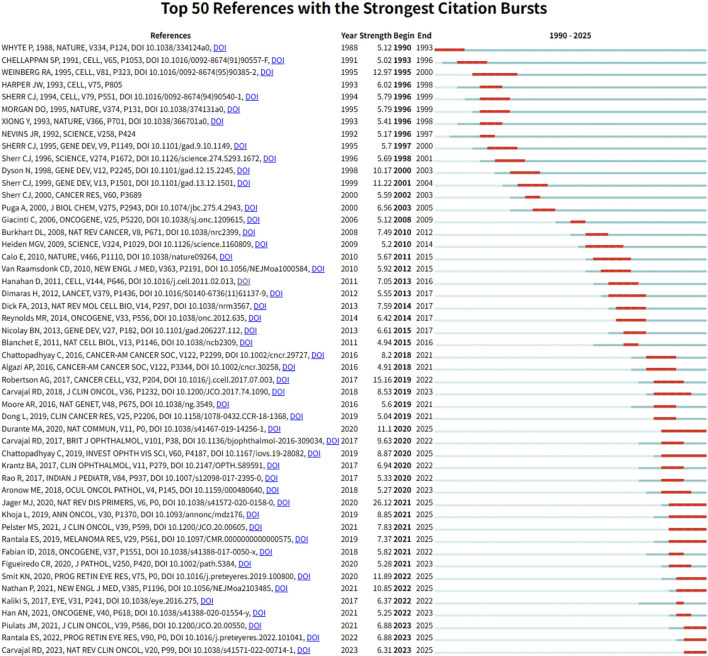
Top 50 references with the strongest citation bursts between 1990 and 2025.

To capture emerging trends and research frontiers in the field of intraocular malignancies and metabolism, we conducted burst detection analysis using CiteSpace. The top 50 references with the strongest citation bursts from 1990 to 2025 are presented in Figure 5. Notably, the most cited reference is the Cancer Cell article titled “Integrative Analysis Identifies Four Molecular and Clinical Subsets in Uveal Melanoma”, which we previously analyzed in detail. This reference reflects a pivotal shift toward molecular subtyping and metabolic profiling in UM research. Among the top 50, 11 references remain in their burst period as of 2025, indicating their continued relevance and influence in shaping future investigations.

**FIGURE 6:**
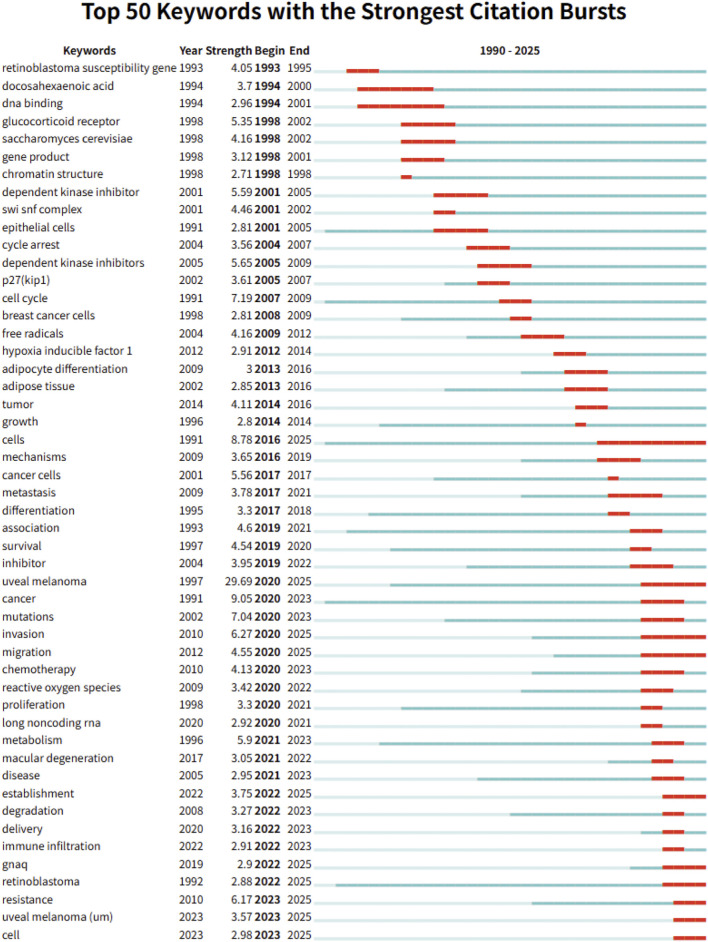
Keywords burst analysis in metabolism related to intraocular tumors.

Complementarily, Figure 6 displays the top 50 keywords with the strongest citation bursts from a total of 698 terms detected. These keywords are indicative of research hotspots and emerging topics in the field. Early-stage bursts included fundamental terms such as “retinoblastoma susceptibility gene” (1993–1995) and “dna binding” (1994–2001), reflecting foundational molecular biology studies. In contrast, more recent bursts—such as “uveal melanoma” (2020-2025, strength: 29.69), “mutations”, “proliferation”, “metabolism”, “immune infiltration”, “gnaq”, “resistance”, and “long noncoding RNA”—demonstrate a growing focus on tumor heterogeneity, metabolic reprogramming, immunological contexture, and biomarker development. Several keywords that emerged after 2022, including “retinoblastoma”, “uveal melanoma”, and “cell”, suggest a revitalized interest in classical entities under new molecular frameworks.

The overlap between recently emerged keywords and ongoing citation bursts of core references underscores a consistent research trajectory: from genomic and transcriptomic classification to metabolic and immune-targeted approaches in intraocular malignancies. These findings point to the sustained and evolving significance of metabolic mechanisms in the study of eye tumors.

### Cross-database validation of results

To assess the robustness of the WoSCC-based findings, equivalent searches were conducted in Scopus and PubMed, yielding 1,980 and 1,520 publications, respectively, within the defined time frame and inclusion criteria. While absolute publication counts varied due to differences in database coverage, the temporal trends of annual publications were highly consistent across the three databases, with Pearson correlation coefficients of 0.962 (WoSCC vs. Scopus) and 0.948 (WoSCC vs. PubMed), both statistically significant (p < 0.001).

The thematic distribution of high-frequency keywords also showed substantial overlap. For instance, terms such as uveal melanoma, retinoblastoma, oxidative stress, apoptosis, and metabolism appeared in the top 10 for all three datasets, indicating concordance in major research focuses. Similarly, the top contributing countries remained unchanged across databases, with the United States and China consistently occupying the first and second ranks in publication volume. Uveal melanoma was identified as the predominant intraocular malignancy in metabolism-related research in all three datasets, reinforcing the primary conclusion derived from the WoSCC analysis.

Minor variations were observed in lower-ranked keywords and country lists, primarily reflecting database-specific indexing policies and journal coverage. However, no substantive differences emerged in the overall trajectory, thematic priorities, or geographical distribution of research. These results confirm that the observed trends, key contributors, and thematic structures are not artifacts of a single database, but rather represent a stable and reproducible pattern across major bibliographic platforms.

## Discussion

This bibliometric analysis offers a thorough examination of global research trends, thematic developments, and collaborative networks in the domain of metabolism-related intraocular malignancies over the past 35 years. The analysis demonstrates a consistent and sustained increase in research activity since 1990, with a notable escalation in publication output and citation frequency post-2010, suggesting an intensifying academic and clinical focus on the metabolic aspects of ocular tumors. Among intraocular malignancies, UM has emerged as the most extensively studied condition, prominently featuring in high-frequency keywords, co-citation clusters, and citation burst references. This prominence underscores both the clinical severity and biological complexity of UM, particularly its links to metabolic dysregulation and therapeutic resistance.

The examination of author affiliations and contributions at the country level underscores the global distribution of research activities, albeit with significant geographic disparities. The United States has consistently held a leading position in terms of both research productivity and academic influence. However, recent years have witnessed a marked increase in contributions from China, although these contributions tend to have a comparatively lower average citation impact and less centrality in international collaboration networks. Author collaboration analysis further reveals that several high-output Chinese research teams form well-defined domestic clusters with strong internal cohesion but relatively weak links to international counterparts. This pattern may reflect structural challenges such as language barriers, limited access to international consortia, differences in research funding frameworks, or institutional incentives that prioritize domestic rather than global engagement. Nevertheless, the increasing convergence of research interests—particularly around shared themes such as immune–metabolic interactions, biomarker discovery, and advanced metabolomic profiling—presents clear opportunities to foster cross-national collaboration. Strengthening such global integration will be critical for advancing translational impact and ensuring the broad applicability of findings in metabolism-related ocular oncology.

Metabolic dysregulation is increasingly recognized as both a hallmark of cancer and a therapeutic vulnerability. Although ocular oncology historically prioritized histopathological classification and local control strategies, our findings demonstrate a clear transition toward mechanism-oriented investigations, particularly those centered on metabolic remodeling. The co-occurrence of keywords such as “glycolysis,” “oxidative phosphorylation,” “lipid metabolism,” and “hypoxia” across multiple clusters underscores the growing focus on the diverse metabolic adaptations employed by intraocular tumors to support proliferation, invasion, and immune evasion. These findings suggest that ocular tumors, despite their anatomical and immunological uniqueness, share many of the metabolic features observed in systemic malignancies. Notably, research on oxidative stress, energy metabolism, and ROS-related signaling has become increasingly prominent, reflecting their central role in intraocular tumor pathophysiology. For instance, in UM, disruptions in mitochondrial respiration and the activation of hypoxia-inducible factors (HIFs) have been implicated in promoting angiogenesis, metastasis, and resistance to therapy (Hutchinson, 2017; Ortega et al., 2020; Sun et al., 2025). Similarly, studies on retinoblastoma have revealed altered glucose metabolism and lactate accumulation as key features of tumor survival within the ocular microenvironment (Mouriaux et al., 2014; Tang et al., 2024). The keyword trajectory also indicates an emerging interest in metabolomics, metabolic biomarkers, and pathway enrichment analysis, signaling a methodological evolution from single-gene explorations to systems-level metabolic profiling.

Within the spectrum of intraocular malignancies analyzed in this study, UM consistently emerged as the most extensively investigated tumor type in the context of metabolism-related research. It appeared as a high-frequency keyword, a core theme in co-citation clusters, and the primary disease focus in the most cited references. This convergence across bibliometric layers highlights UM is a representative model for investigating metabolic reprogramming, molecular heterogeneity, and translational innovation in ocular oncology. At the molecular level, UM is distinguished by a relatively low mutational burden, yet it harbors recurrent mutations in pivotal driver genes, such as GNAQ, GNA11, and BAP1. These mutations are intricately linked to downstream alterations in oxidative metabolism, mitochondrial function, and lipid remodeling (Schadendorf et al., 2015; Cunanan et al., 2025). For instance, the activation of the MAPK and YAP pathways mediated by GNAQ/GNA11 has been demonstrated to reprogram glucose and fatty acid utilization in UM cells, thereby enhancing survival under nutrient-deprived or hypoxic conditions (Zhang et al., 2018; Han et al., 2023). Concurrently, the inactivation of BAP1, which is strongly associated with an elevated risk of metastasis, is connected to extensive epigenetic and metabolic reprogramming, including impaired oxidative phosphorylation and altered lipid droplet formation (Bustamante et al., 2021). These findings indicate that the metabolic phenotype of UM is not merely a secondary effect of oncogenic signaling but constitutes an integral component of its malignant progression and immune evasion. From a clinical perspective, the propensity of UM for hematogenous metastasis to the liver—a metabolically distinct and immunologically specialized organ—underscores the importance of metabolic research in this area. The hepatic microenvironment imposes unique selective pressures on metastatic UM cells, promoting the survival of clones that can adapt to oxidative stress, lipid-rich conditions, and immune suppression. Recent studies (Gong et al., 2024; Wang et al., 2025) have identified the accumulation of lactate, the modulation of reactive oxygen species (ROS), and the exploitation of tryptophan-kynurenine metabolism as potential mechanisms by which UM cells evade immune surveillance and proliferate in the liver. These findings reflect an increasing recognition of the interplay between metabolism and the tumor immune microenvironment (TME), positioning UM as a valuable model for studying metabolic-immunologic interactions. Collectively, the metabolic landscape of UM provides a rich and multifaceted platform for investigating fundamental cancer biology and developing therapeutic strategies that target or are informed by metabolic processes. The integration of metabolic profiling with genetic subtyping, immune contexture analysis, and response prediction models is likely to pave the way for precision oncology approaches tailored to UM’s unique biology.

The progression of keyword trends and co-citation patterns in our analysis underscores the increasing integration of multi-omics methodologies into metabolism-focused investigations of intraocular malignancies. This trend highlights the growing acknowledgment that tumor metabolism is intricately interconnected with genomic, epigenomic, transcriptomic, and immune landscapes, thereby necessitating comprehensive analytical frameworks to fully elucidate its complexity. Notably, the convergence of metabolomics with transcriptomic and single-cell sequencing technologies is beginning to yield unprecedented insights into the metabolic heterogeneity of ocular tumors. In the context of UM, recent studies (Dewaele et al., 2022; Zhan et al., 2024) have combined RNA sequencing with metabolic pathway analysis to identify gene expression signatures linked to mitochondrial dysfunction, lipid metabolism, and oxidative phosphorylation dysregulation factors that are intimately associated with tumor progression and immune evasion. In the context of retinoblastoma, the integration of single-cell RNA sequencing (scRNA-seq) with metabolic flux analysis has elucidated cell-type-specific metabolic alterations, notably the upregulation of glycolysis and the downregulation of oxidative phosphorylation in proliferating tumor cells (Tang et al., 2024; Zhang et al., 2025). These insights underscore the value of multi-omics approaches in pinpointing subtype-specific metabolic vulnerabilities, which could inform precision treatment strategies. Furthermore, our bibliometric analysis indicates a growing adoption of technologies such as nuclear magnetic resonance (NMR) spectroscopy and liquid chromatography-mass spectrometry (LC-MS) as fundamental tools in ocular oncology research. These platforms facilitate quantitative and high-throughput metabolite profiling, advancing the field from descriptive studies to mechanistic explorations and the identification of clinically actionable metabolic biomarkers. For instance, NMR-based metabolomics has been employed to differentiate between metastatic and non-metastatic UM based on serum and aqueous humor profiles, while LC-MS has identified lipidomic signatures correlated with BAP1 mutation status and prognosis (Gulati et al., 2023). With the increasing accessibility and standardization of these techniques, it is anticipated that the divide between laboratory-based metabolic research and practical clinical diagnostics will be narrowed. Collectively, the swift advancement of omics-enabled metabolic research signifies a comprehensive transformation in the study of intraocular tumors, transitioning from reductionist models to integrative, data-driven scientific approaches. This paradigm shift not only deepens our mechanistic comprehension of ocular tumor biology but also hastens the translation of metabolic insights into innovative diagnostic, prognostic, and therapeutic strategies.

The evolving landscape of metabolism-related research in intraocular malignancies presents promising opportunities for advancements in early diagnosis, therapeutic innovation, and personalized treatment strategies. An analysis of emergent keywords and highly cited literature indicates several pivotal directions likely to influence the future trajectory of this field. Firstly, the identification and clinical validation of metabolic biomarkers are of considerable interest for early detection and disease monitoring. Metabolomic profiling of intraocular fluids, such as aqueous humor and vitreous samples, has already uncovered tumor-specific metabolic signatures. This discovery suggests that minimally invasive diagnostic tools based on metabolite panels could significantly enhance early-stage diagnosis and recurrence surveillance, particularly in cases of retinoblastoma and UM (Dyer, 2016; Yang et al., 2021). This trend is reflected in the recent emergence of keywords such as “metabolomics,” “NMR spectroscopy,” and “LC-MS,” highlighting growing methodological emphasis on metabolic biomarker discovery. Secondly, there is growing interest in targeting metabolic signaling pathways, including PI3K/AKT/mTOR, AMPK, and HIF-1α, as a therapeutic approach to disrupt the metabolic dependencies of ocular tumors. Preclinical studies have shown that pharmacological inhibition of these pathways can suppress tumor growth, increase radiosensitivity, and modulate angiogenesis. The prominence of keywords such as “hypoxia,” “oxidative phosphorylation,” and “glycolysis” in our co-occurrence and clustering analyses further supports the centrality of these metabolic pathways in the current research landscape. The intersection of these pathways with oncogenic drivers and immune regulators underscores the rationale for developing combination strategies tailored to the metabolic genotype and immune phenotype of individual tumors. Furthermore, the integration of metabolic interventions with immunomodulatory therapies presents a promising translational avenue. Emerging evidence indicates that metabolic reprogramming within the TME—characterized by lactate accumulation, oxygen depletion, and ROS imbalance—can suppress anti-tumor immunity. Modulating metabolic checkpoints to restore immune activity, such as through the inhibition of lactate production, stabilization of HIF-1α, or blockade of tryptophan metabolism, may counteract immune evasion and enhance the efficacy of checkpoint blockade or adoptive cell therapy in UM(Jiang et al., 2024; Zheng et al., 2024). This direction is strongly echoed by recent burst keywords such as “immune infiltration,” “tryptophan metabolism,” and “ROS,” reflecting the field’s increasing attention to the metabolism–immunity interface.

Several limitations should be acknowledged. First, the search was limited to English language publications, which may have excluded relevant non-English studies and introduced language bias. Second, citation-based comparisons across countries and institutions may be influenced by differences in publication timing, while we report raw citation counts and average citation ratios, we acknowledge the absence of field-normalized metrics or fixed citation windows. Third, the results of co-occurrence, clustering, and citation burst analyses are inherently influenced by the selected algorithms and thresholds, which involve a degree of semantic and methodological subjectivity (Wu et al., 2023). Lastly, while bibliometric indicators such as citation counts, h-index, and betweenness centrality offer valuable insights into academic influence and network positioning, they do not directly reflect the quality, innovation, or translational impact of individual studies.

While metabolic research in intraocular tumors is not as advanced as in other oncologic subspecialties, the significant increase in publication volume and thematic diversification over the past decade indicates its emergence as a dynamic and promising research frontier. The growing integration of metabolic insights with precision oncology, studies of the immune microenvironment, and the development of targeted therapies highlights the translational significance of this field. As novel therapeutic strategies—such as inhibitors of oxidative metabolism, modulators of lipid pathways, and metabolic–immunologic co-targeting approaches—advance into preclinical and clinical evaluation, the continued expansion of this research domain is poised to transform the diagnostic and therapeutic landscape of ocular oncology.

## Conclusion

This study offers a comprehensive bibliometric and visualized analysis of metabolism-related research in intraocular malignancies from 1990 to 2025, highlighting evolving publication trends, influential contributors, and emerging thematic hotspots. This analysis is expected to guide future scientific exploration and support the development of innovative, metabolism-based strategies for the diagnosis and treatment of intraocular tumors.

## Data Availability

The original contributions presented in the study are included in the article/Supplementary Material, further inquiries can be directed to the corresponding authors.
