# Supplementary material for: Global research trends in metabolism-related intraocular malignancies: a multi-database bibliometric analysis and cross-validation study
Source: Front Mol Biosci. 2025 Sep 9;12:1683864. doi: 10.3389/fmolb.2025.1683864 (PMC12454098; doi:10.3389/fmolb.2025.1683864)
Supplement: Supplementary file 1 [file Supplementaryfile1.docx]

**Supplementary Table S1. The complete search formula.**

TS=("Retinoblastoma*" OR "Retinal Glioblastoma*" OR "Retinal Glioma*" OR "Retinal Neuroblastoma*" OR "Uveal Melanoma*" OR "Intraocular Melanoma*" OR "Ocular Melanoma*" OR "Choroidal Melanoma*" OR "Iris Melanoma*" OR "Ciliary Body Melanoma*" OR "Ciliochoroidal Melanoma*" OR "Choroid Neoplasm*" OR "Choroidal Neoplasm*" OR "choroidal metastasis*" OR "choroidal metastases*" OR "metastatic intraocular tumor" OR "Intraocular Lymphoma" "primary vitreoretinal lymphoma" OR "ocular lymphoma" OR "choroidal lymphoma" OR "retinal melanoma" OR "primary retinal melanoma" OR "intraocular sarcoma" OR "intraocular sarcomas" OR "retinal sarcoma" OR "intraocular malignancy" OR "intraocular malignant tumor" OR "intraocular malignant neoplasm" OR "choroidal malignancy" OR "retinal malignancy") AND TS=(metabolism OR "metabolic reprogramming" OR "metabolic alterations" OR "metabolic rewiring" OR "energy metabolism" OR "cellular metabolism" OR "tumor metabolism" OR "oncometabolism" OR "glucose metabolism" OR "aerobic glycolysis" OR "glycolysis" OR "glycolytic activity" OR "oxidative phosphorylation" OR "OXPHOS" OR "mitochondrial metabolism" OR "mitochondrial dysfunction" OR "fatty acid metabolism" OR "lipid metabolism" OR "lipogenesis" OR "lipolysis" OR "amino acid metabolism" OR "glutamine metabolism" OR "serine metabolism" OR "lactate" OR "glucose" OR "ATP" OR "pyruvate" OR "succinate" OR "citrate" OR "NADH" OR "ROS" OR "Warburg effect" OR "reverse Warburg effect" OR "hypoxia" OR "HIF-1α" OR "oxidative stress" OR "metabolite profiling" OR "metabolic fingerprinting" OR "metabolic pathway enrichment" OR "metabolomics" OR "metabonomics" OR "untargeted metabolomics" OR "targeted metabolomics" OR "LC-MS" OR "GC-MS" OR "NMR spectroscopy" OR "mass spectrometry-based metabolomics" OR "metabolic biomarkers" OR "bioenergetics" OR "metabolic flux" OR "pathway enrichment analysis" OR "KEGG pathway" OR "Reactome metabolism" OR "metabolic signature" OR "metabolic phenotyping")

**Supplementary Table S2. The top 10 most productive countries.**

| **Rank** | **Country/region** | **Article counts** | **centrality** | **Percentage (%)** | **Citation** | **Citation per publication** |
| --- | --- | --- | --- | --- | --- | --- |
| **1** | USA | 666 | 0.47 | 38.17% | 48850 | 73.35 |
| **2** | CHINA | 389 | 0.1 | 22.29% | 8457 | 21.74 |
| **3** | GERMANY | 111 | 0.05 | 6.36% | 5072 | 45.69 |
| **4** | JAPAN | 101 | 0.02 | 5.79% | 5679 | 56.23 |
| **5** | CANADA | 96 | 0.06 | 5.50% | 5756 | 59.96 |
| **6** | ITALY | 85 | 0.18 | 4.87% | 3897 | 45.85 |
| **7** | ENGLAND | 78 | 0.08 | 4.47% | 6315 | 80.96 |
| **8** | SPAIN | 74 | 0.17 | 4.24% | 4199 | 56.74 |
| **9** | INDIA | 72 | 0.07 | 4.13% | 1576 | 21.89 |
| **10** | FRANCE | 62 | 0.14 | 3.55% | 3670 | 59.19 |

**Supplementary Table S3. The top 10 institutions publishing literature.**

| **Rank** | **Institution** | **Country** | **Number of studies** | **Total citations** | **Average citation** |
| --- | --- | --- | --- | --- | --- |
| **1** | Harvard University | USA | 57 | 5208 | 91.37 |
| **2** | Harvard University Medical Affiliates | USA | 50 | 4365 | 87.30 |
| **3** | University of California System | USA | 33 | 3380 | 102.42 |
| **4** | Shanghai Jiao Tong University | China | 33 | 1207 | 36.58 |
| **5** | Pennsylvania Commonwealth System of Higher Education (PCSHE) | USA | 32 | 2529 | 79.03 |
| **6** | University of Texas System | USA | 31 | 1963 | 63.32 |
| **7** | University System of Ohio | USA | 27 | 2365 | 87.59 |
| **8** | Institut National de la Sante et de la Recherche Medicale (Inserm) | France | 26 | 1321 | 50.81 |
| **9** | Chinese Academy of Sciences | China | 26 | 989 | 38.04 |
| **10** | National Institutes of Health (NIH) - USA | USA | 25 | 1537 | 61.48 |

**Supplementary Table S4. The top 10 productive journals.**

| **Rank** | **Journal** | **Article counts** | **Percentage(1745)** | **IF** | **Quartile in category** |
| --- | --- | --- | --- | --- | --- |
| **1** | journal of biological chemistry | 66 | 3.78% | 3.9 | Q2 |
| **2** | oncogene | 38 | 2.18% | 7.3 | Q1 |
| **3** | international journal of molecular sciences | 33 | 1.89% | 4.9 | Q1 |
| **4** | plos one | 33 | 1.89% | 2.6 | Q2 |
| **5** | investigative ophthalmology & visual science | 32 | 1.83% | 4.7 | Q1 |
| **6** | cancer research | 30 | 1.72% | 16.6 | Q1 |
| **7** | cancers | 30 | 1.72% | 4.4 | Q2 |
| **8** | molecular and cellular biology | 21 | 1.20% | 2.7 | Q3 |
| **9** | experimental eye research | 18 | 1.03% | 2.7 | Q2 |
| **10** | biochemical and biophysical research communications | 16 | 0.92% | 2.2 | Q3 |

**Supplementary Table S5. The top 10 co-cited journals.**

| **Rank** | **Cited Journal** | **Co-Citation** | **IF(2024)** | **Quartile in category** |
| --- | --- | --- | --- | --- |
| **1** | P NATL ACAD SCI USA | 1076 | 9.1 | Q1 |
| **2** | J BIOL CHEM | 1045 | 3.9 | Q2 |
| **3** | NATURE | 1031 | 48.5 | Q1 |
| **4** | CELL | 939 | 42.5 | Q1 |
| **5** | CANCER RES | 938 | 16.6 | Q1 |
| **6** | ONCOGENE | 869 | 7.3 | Q1 |
| **7** | SCIENCE | 839 | 45.8 | Q1 |
| **8** | MOL CELL BIOL | 695 | 2.7 | Q3 |
| **9** | GENE DEV | 632 | 7.7 | Q1 |
| **10** | PLOS ONE | 532 | 2.6 | Q2 |

**Supplementary Table S6. Top 10 most prolific and co-cited authors in the field of metabolism related to intraocular tumors.**

| **Rank** | **Author** | **Count** | **Rank** | **Co-cited author** | **Citation** |
| --- | --- | --- | --- | --- | --- |
| **1** | Fan, xianqun | 16 | 1 | Sherr cj | 189 |
| **2** | Ge, shengfang | 12 | 2 | Harbour jw | 126 |
| **3** | Jia, renbing | 12 | 3 | Weinberg ra | 124 |
| **4** | Jager, martine j. | 10 | 4 | Shields cl | 96 |
| **5** | Krishnakumar, subramanian | 10 | 5 | Singh ad | 84 |
| **6** | Murray, timothy g. | 9 | 6 | Carvajal rd | 69 |
| **7** | Zhuang, ai | 9 | 7 | Dimaras h | 66 |
| **8** | Cree, ia | 8 | 8 | Jager mj | 64 |
| **9** | Kakkassery, vinodh | 8 | 9 | Dyson n | 60 |
| **10** | Palou, andreu | 8 | 10 | Chattopadhyay c | 56 |

**Supplementary Table S7. Top 10 co-cited references with the highest centrality.**

| **Rank** | **Title** | **Journal** | **Total citations** |
| --- | --- | --- | --- |
| **1** | Integrative Analysis Identifies Four Molecular and Clinical Subsets in Uveal Melanoma | CANCER CELL | 30 |
| **2** | Single-cell analysis reveals new evolutionary complexity in uveal melanoma | NAT COMMUN | 25 |
| **3** | The retinoblastoma protein and cell cycle control | CELL | 24 |
| **4** | Uveal melanoma: Towards a molecular understanding | PROG RETIN EYE RES | 23 |
| **5** | Overall Survival Benefit with Tebentafusp in Metastatic Uveal Melanoma | NEW ENGL J MED | 21 |
| **6** | Elevated Endogenous SDHA Drives Pathological Metabolism in Highly Metastatic Uveal Melanoma | INVEST OPHTH VIS SCI | 20 |
| **7** | CDK inhibitors: positive and negative regulators of G1-phase progression | GENE DEV | 20 |
| **8** | Meta-analysis in metastatic uveal melanoma to determine progression free and overall survival benchmarks: an international rare cancers initiative (IRCI) ocular melanoma study | ANN ONCOL | 18 |
| **9** | Metastatic disease from uveal melanoma: treatment options and future prospects | BRIT J OPHTHALMOL | 18 |
| **10** | The regulation of E2F by pRB-family proteins | GENE DEV | 18 |

**Supplementary Table S8. Top 20 most frequent and central keywords in metabolism related to intraocular tumors.**

| **Rank** | **Keyword** | **Counts** | **Rank** | **Keyword** | **Counts** |
| --- | --- | --- | --- | --- | --- |
| **1** | retinoblastoma protein | 320 | 11 | phosphorylation | 100 |
| **2** | apoptosis | 236 | 12 | survival | 92 |
| **3** | uveal melanoma | 226 | 13 | inhibition | 80 |
| **4** | oxidative stress | 211 | 14 | cell-cycle | 75 |
| **5** | metabolism | 139 | 15 | progression | 74 |
| **6** | proliferation | 132 | 16 | differentiation | 71 |
| **7** | activation | 130 | 17 | pathway | 67 |
| **8** | protein | 117 | 18 | angiogenesis | 65 |
| **9** | hypoxia | 102 | 19 | transcription | 62 |
| **10** | p53 | 100 | 20 | mutations | 59 |
